# Multigene phylogenetic analyses to establish new *Valsaria* species and taxonomic significance of spore ornamentation

**DOI:** 10.1371/journal.pone.0217982

**Published:** 2019-06-26

**Authors:** Dhandevi Pem, Kevin D. Hyde, Mingkwan Doilom, Erio Camporesi, Sinang Hongsanan, Sillma Rampadarath, Vishwakalyan Bhoyroo, Rajesh Jeewon

**Affiliations:** 1 Key Laboratory for Plant Diversity and Biogeography of East Asia, Kunming Institute of Botany, Chinese Academy of Science, Kunming, Yunnan, People’s Republic of China; 2 Center of Excellence in Fungal Research, Mae Fah Luang University, Chiang Rai, Thailand; 3 Mushroom Research Foundation, 128 M.3 Ban Pa Deng T. Pa Pae, A. Mae Taeng, Chiang Mai, Thailand; 4 World Agroforestry Centre, East and Central Asia, Kunming, Yunnan, P. R. China; 5 A.M.B. Gruppo Micologico Forlivese “Antonio Cicognani”, Via Roma, Forlì, Italy; 6 A.M.B. Circolo Micologico “Giovanni Carini”, Brescia, Italy; 7 Società per gli Studi Naturalistici della Romagna, Bagnacavallo (RA), Italy; 8 Shenzhen Key Laboratory of Microbial Genetic Engineering, College of Life Sciences and Oceanography, Shenzhen University, Shenzhen, China; 9 Faculty of Agriculture, University of Mauritius, Reduit, Mauritius; 10 Department of Health Sciences, Faculty of Science, University of Mauritius, Reduit, Mauritius; Tianjin University, CHINA

## Abstract

During our studies on fungal diversity from plant substrates, a new species of *Valsaria* was isolated from dead branches of *Ostrya carpinifolia*. The taxon is morphologically similar to other taxa in Valsariaceae and is characterized by pseudostromata, apically free pseudoparaphyses, bitunicate asci, and dark brown, 2-celled ascospores. However, it differs from extant species in number of guttules and ornamentation of spore. It is introduced herein as *Valsaria ostryae* sp. nov. within the family Valsariaceae. Multigene phylogenies based on combined LSU, ITS and RPB2 DNA sequence data generated from maximum likelihood, maximum parsimony and MrBayes analyses indicate that *V*. *ostryae* is basal to *V*. *lopadostomoides* and *V*. *rudis* and its establishment as a new species is strongly supported. No discordance was found between our morphological and phylogenetic species boundaries as postulated by other researchers and our molecular data strongly supports ornamentation of spore as useful for species delineation. *Valsaria* species do not appear to be host specific. Full morphological details are provided herein and phylogenetic relationships of *Valsaria* species are also discussed in light with host association.

## Introduction

Valsariaceae was introduced by Jaklitsch et al. [1] and classified in the order Valsariales. Species of this family have a worldwide distribution occurring on sun-exposed, corticated logs, branches of coniferous and broadleaf trees and on bamboo [1] and exist as saprobes, plant pathogens or necrotrophs [2,3]. The family comprises three genera namely *Bambusaria* Jaklitsch et al., *Myrmaecium* Nitschke ex Fuckel and *Valsaria* Ces. & De Not [1,4]. The asexual morphs are known to be coelomycetous [4]. *Valsaria* was introduced by Cesati and De Notaris [5] with *Valsaria insitiva* (Tode) Ces. & De Not. as the type species. Ju et al. [2] examined numerous species to provide an update of its taxonomy. Multigene phylogenetic analyses have also been performed to clarify species taxonomy [1]. In this paper, we introduce one new species of *Valsaria*, which was found on dead terrestrial branches of *Ostrya carpinifolia* (Betulaceae). Combined analyses of LSU, ITS and RPB2 DNA sequence data using maximum-likelihood (ML), maximum-parsimony (MP) and Bayesian inference analyses clearly indicate that this species belongs to the Valsariaceae with strong statistical support. Phylogeny also provides important clues on the taxonomic usefulness of morphological features that can be used to differentiate among *Valsaria* species and its allies. In this paper, the new species is described, illustrated and compared with similar taxa.

## Materials and methods

### Isolates and morphology

**Fig 1:**
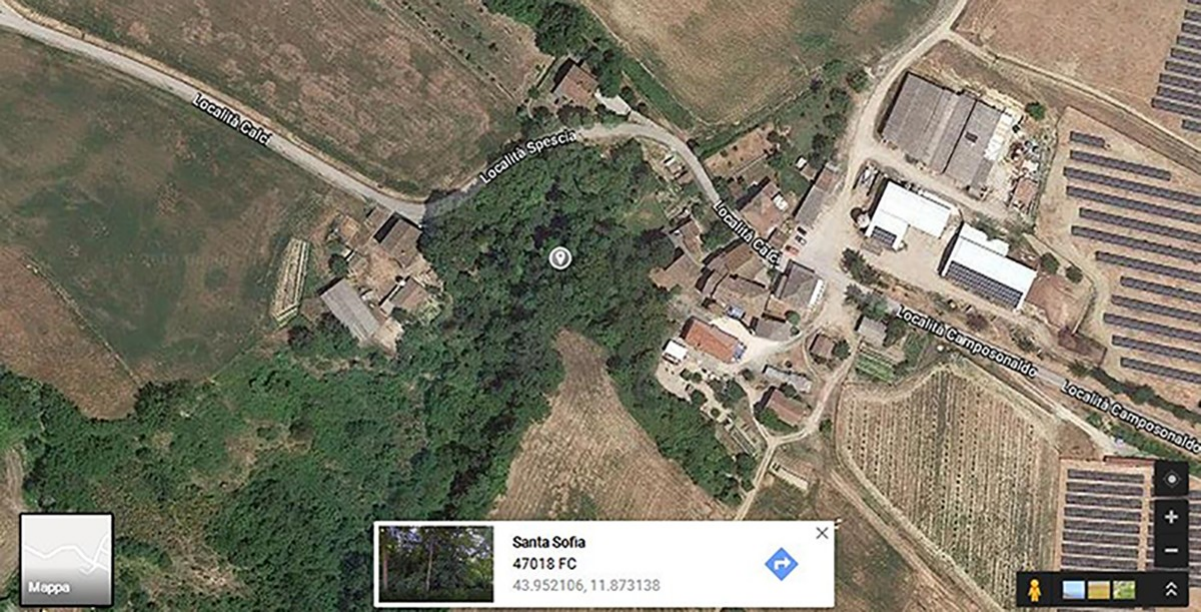
Geographical coordinates of sample collected.

Specimens were collected from dead branches of *Ostrya carpinifolia* in the Province of Forlì-Cesena [FC], Santa Sofia, Camposonaldo, Italy and were pressed and dried individually between blotting papers and labelled. The geographic coordinates for the data collected is 43.952106, 11.873138 (Fig 1). Specimens were observed under a Motic SMZ 168 series dissecting stereo-microscope. Hand-cut sections of the fruiting structures were mounted in water for microscopic studies and photomicrography. Pure cultures were obtained from single spore isolation following the method designated in Chomnunti et al. [6]. Germinating ascospores were transferred aseptically to malt extract agar (MEA) plates and incubated at 16°C. Colony characters on media were observed and growth rates were measured after one week and at weekly intervals. No specific permissions were required for the location because permissions are standardized only for macro-fungi such as only 3 fruiting-bodies for each collection, the indication of the name of the species that it needs to collect and other things valid only for macrofungi. Also, the field studies did not involve endangered or protected species. The holotype specimen was deposited in the Mae Fah Luang University Herbarium (MFLU), Chiang Rai, Thailand. Ex-type living cultures are deposited in the Culture Collection at Mae Fah Luang University (MFLUCC) and at the International Collection of Microorganisms from Plants (ICMP). Faces of Fungi and Index Fungorum numbers are provided as in Jayasiri et al. [7] and Index Fungorum [8]. New taxa were established based on recommendations outlined by Jeewon and Hyde [9].

### DNA extraction, PCR amplification and sequencing

Fungal isolates were grown on MEA media at 16 ± 2°C for 4 weeks. Fungal mycelium was extracted with Biospin Fungus Genomic DNA Extraction Kit-BSC14S1 (BioFlux, P.R. China). Amplification reactions were performed using known primer pairs, LR0R/LR5 for the 28S large subunit rDNA (LSU) [10], ITS5/ITS4 for 5.8S nrRNA gene with the two flanking internal transcribed spacers (ITS) [11] and fRPB2-5F/ fRPB2-7cR for RNA polymerase II second largest subunit (RPB2) [12]. The ultimate volume of the PCR reaction were 25 μl, containing 1 μl of DNA template (40 μg), 1 μl of each forward and reverse primer (0.2 μM) and 12.5 μl of 2xMaster Mix and 9.5 μl of ddH2O. The PCR condition was as follows: initial denaturation 95°C for 3 min, 35 cycles of denaturation at 95°C for 30 s, annealing at 54°C for 48 s, elongation at 72°C for 1 min, and final extension at 72°C for 10 min for LSU, ITS and RPB2. PCR products were sequenced with the same primers mentioned above at Sangon Biotech, Shanghai, China.

### Sequence alignment and phylogenetic analyses

**Fig 2:**
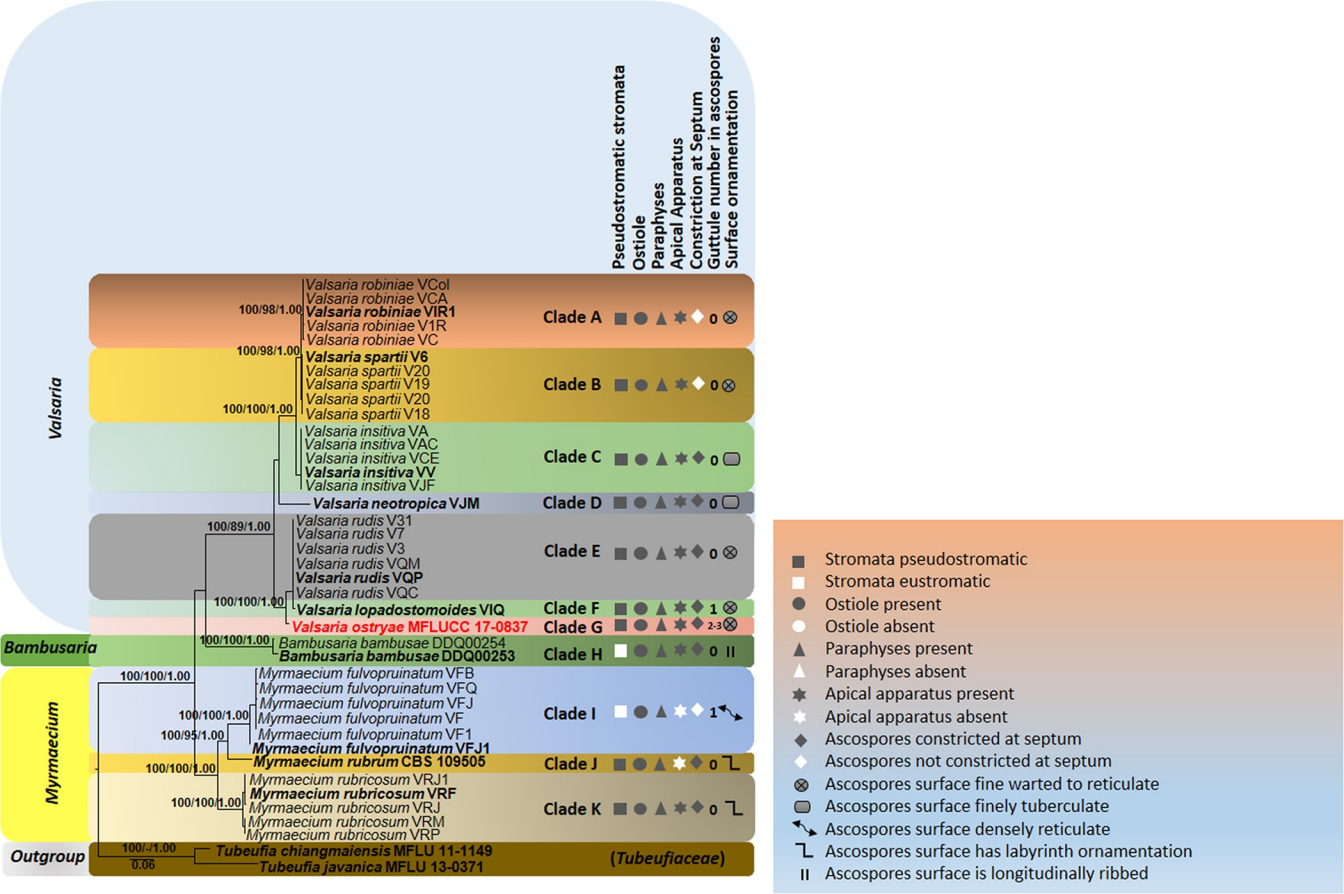
RAxML tree based on DNA sequence analyses of a combined dataset of LSU, ITS and RPB2 gene regions. Bootstrap support values for ML and MP equal to or greater than 90%, Bayesian posterior probabilities (PP) equal to or greater than 0.90 are defined as ML/MP/PP above or below the nodes. Taxonomic novelty is indicated in red and ex-type strains are in bold text. GenBank accession numbers are indicated at the end of the species name. The tree was rooted to *Tubeufia chiangmaiensis* MFLU 11–1149, *Tubeufia javanica* MFLU 13–0371.

Phylogenetic analyses were conducted on a combined dataset of LSU, ITS and RPB2 sequence data. Taxa used in the analyses were obtained through recent publications [1, 13]. Separate LSU, ITS and RPB2 DNA sequences were also subjected to BLAST search engine tool of NCBI for verification and selection of taxa for subsequent phylogenetic analyses. Maximum likelihood analysis was performed by using raxmlGUIv.0.9b2 [14]. The search strategy was set to rapid bootstrapping and the analysis carried out using the GTRGAMMAI model of nucleotide substitution. The number of replicates was inferred using the stopping criterion [15]. Maximum Likelihood bootstrap values equal or greater than 90% are given as the first set of numbers above the nodes (Fig 2). PAUPv4.0b10 was used to conduct the parsimony analysis to obtain the phylogenetic trees. Trees were inferred using the heuristic search option with 1000 random sequence additions. Maxtrees were setup to 500 and branches of zero length were collapsed and all multiple parsimonious trees were saved. Descriptive tree statistics for parsimony (Tree Length [TL], Consistency Index [CI], Retention Index [RI], Relative Consistency Index [RC] and Homoplasy Index [HI] were calculated for trees generated under different optimality criteria. Kishino-Hasegawa tests (KHT) [16] were performed in order to determine whether trees were significantly different. Maximum-parsimony bootstrap values equal or greater than 90% are given as the second set of numbers above the nodes (Fig 2). Bayesian Inference (BI) analysis was conducted with MrBayes v. 3.1.2 [17] to evaluate Posterior probabilities (BYPP) (Rannala and Yang 1996, Zhaxybayeva and Gogarten 2002) by Markov Chain Monte Carlo sampling (BMCMC). Two parallel runs were conducted, using the default settings, but with the following adjustments: Six simultaneous Markov chains were run for 5,000,000 generations and trees were sampled every 1000 generation. The distribution of log-likelihood scores was examined to determine stationary phase for each search and to decide if extra runs were required to achieve convergence, using the program Tracer 1.5 [18]. First 20% of generated trees were discarded and remaining 80% of trees were used to calculate posterior probabilities (PP) of the majority rule consensus tree. Phylograms were visualized with FigTree v1.4.0 program [19] and reorganized in Microsoft power point (2016) and Adobe Illustrator CS5 (Version 15.0.0, Adobe, San Jose, CA). The finalized alignment and tree is deposited in TreeBASE, submission ID: 22749 (http://purl.org/phylo/treebase/phylows/study/TB2:S22749).

### Nomenclature

The electronic version of this article in Portable Document Format (PDF) in a work with an ISSN or ISBN will represent a published work according to the International Code of Nomenclature for algae, fungi, and plants, and hence the new names contained in the electronic publication of a PLOS ONE article are effectively published under that Code from the electronic edition alone, so there is no longer any need to provide printed copies.

In addition, new names contained in this work have been submitted to Index Fungorum from where they will be made available to the Global Names Index. The unique Index Fungorum number can be resolved and the associated information viewed through any standard web browser by appending the Index Fungorum number contained in this publication to the prefix www.indexfungorum.org/. The online version of this work is archived and available from the following digital repositories: PubMed Central, LOCKSS.

### Compliance with ethical standards

There is no conflict of interest (financial or non-financial) and all authors have agreed to submission of paper. The authors also declare that they have no conflict of interest and confirm that the field studies did not involve endangered or protected species.

## Results

### Phylogenetic analyses

The combined LSU, ITS, RPB2 dataset which comprises 38 strains of Valsariaceae species was analyzed to determine the placement of our new taxon and infer relationships at the intrageneric level. The phylogenetic trees obtained from maximum likelihood and parsimony analyses yielded trees with similar overall topology and in agreement with previous study based on maximum likelihood analysis [1]. The maximum parsimony dataset consists of 2414 characters with 1707 characters as constant information, 52 characters as variable characters are parsimony-uninformative, and 655 characters were counted as parsimony informative character. The most parsimonious tree had the following tree scores: TL = 1666, CI = 0.744, RI = 0.930, RC = 0.692, HI = 0.256 values. The best scoring RAxML tree is presented in Fig 2. For better phylogenetic inferences derived from our analyses, we divided the ingroup taxa in the phylogram (Fig 2) into eleven clades (A–K) which corresponds to different *Valsaria* species, including our new taxon. All *V*. *robiniae* strains clustered together in one clade (clade A) with high statistical support (100% ML/ 99% MP/ 1.00 PP). Clade B appearing in 100% ML/ 98% MP/ 1.00 PP of the bootstrap replicates comprises *V*. *spartii* while *V*. *insitiva* strains are basal (clade C) and all these taxa constitute a strongly supported monophyletic clade (100% ML/ 100% MP/ 1.00 PP). *Valsaria neotropica* (clade D) is basal to *V*. *robiniae*, *V*. *spartii* and *V*. *insitiva*, but its relationships to these species receive weak support while *V*. *rudis* (clade E) and *V*. *lopadostomoides* (clade F) cluster together in a monophyletic group but with no support. Our new taxon, *V*. *ostryae* (clade G) is basal to V. *lopadostomoides* and *V*. *rudis*. Individual LSU, ITS and RPB2 single gene trees were generated from individual datasets and phylogenies recovered were essentially topologically similar with no conflicting phylogenetic signals (data not shown). The new sequence data are deposited in GenBank (Table 1).

**Table 1:** Culture collection code and GenBank accession numbers of fungal strains used in this study.

						Genbank Accession No		
Species	Isolate No.	Code	Herbarium No.	Substrate/Host	Country	LSU	ITS	Rpb2
*Bambusaria bambusae*	MFLUCC 12–0851	DDQ00253	MFLU 15–0050	*Thyrsostachys siamensis*	Thailand	KP687812	KP687812	KP687890
*Bambusaria bambusae*	CBS 139763	DDQ00254	MFLU 15–0051	*Thyrsostachys siamensis*	Thailand	KP687813	KP687813	KP687891
*Myrmaecium fulvopruinatum*	CBS 139057	VF	WU 33433	*Fagus sylvatica*	Austria	KP687858	KP687858	KP687933
*Myrmaecium fulvopruinatum*		VF1	WU 33434	*Fraxinus excelsior*, *Alnus glutinosa*	Austria	KP687859	KP687859	KP687934
*Myrmaecium fulvopruinatum*		VFB	WU 33436	*Betula pendula*	Austria	KP687860	KP687860	KP687935
*Myrmaecium fulvopruinatum*	CBS 139058	VFJ	NY (M.E.B.B. 6905)(E)	*Fagus grandifolia*	U.S.A.	KP687861	KP687861	KP687936
*Myrmaecium fulvopruinatum*		VFJ1	WU 33437	unidentified corticated twigs	Taiwan	KP687862	KP687862	KP687937
*Myrmaecium fulvopruinatum*	CBS 139059	VFQ	WU 33438	*Quercus cerris*	Austria	KP687863	KP687863	KP687938
*Myrmaecium rubricosum*	CBS 139067	VRF	WU 33447	unidentified bark	France	KP687881	KP687881	KP687955
*Myrmaecium rubricosum*		VRJ		*Quercus robur*	France	KP687882	KP687882	KP687956
*Myrmaecium rubricosum*		VRJI	WU 33448	unidentified bark	U.S.A.	KP687883	KP687883	KP687957
*Myrmaecium rubricosum*	CBS 139069	VRM	WU 33449	*Picea abies*	Austria	KP687884	KP687884	
*Myrmaecium rubricosum*	CBS 139068	VRP	WU 33450	*Quercus pubescens*	Croatia	KP687885	KP687885	KP687958
*Myrmaecium rubrum*	CBS 109505			*Quercus sp*.	Italy	GU456324	-	GU456344
*Tubeufia chiangmaiensis*	MFLUCC 11–0514	-	MFLU 11–1149	dead wood of an unidentified tree	Thailand	KF301538	KF301530	-
*Tubeufia javanica*	MFLUCC 12–0545	-	MFLU 13–0371,	dead culm-sheath of bamboo	Thailand	KJ880036	KJ880034	-
*Valsaria insitiva*	CBS 139056	VA	WU 33453	*Acer monspessulanum*	Croatia	KP687847	-	KP687922
*Valsaria insitiva*		VAC	WU 33454	*Acer campestre*	Austria	KP687849	KP687849	KP687924
*Valsaria insitiva*		VAF	WU 33455	*Ficus carica*	France	KP687850	KP687850	KP687925
*Valsaria insitiva*		VCE	WU 33456	*Cercis siliquastrum*	Greece	KP687854	KP687854	
*Valsaria insitiva*	CBS 127882	VV	WU 33462 (E)	*Vitis vinifera*	Croatia	KP687886	KP687886	KP687959
*Valsaria lopadostomoides*	CBS 139062	VIQ	WU 33470 (H)	*Quercus ilex*	Greece	KP687868	KP687868	
*Valsaria neotropica*	CBS 139064	VJM	WU 33471 (H)	unidentified corticated twig	France	KP687874	KP687874	KP687948
***Valsaria ostryae***	**MFLUCC 18–1123**		**MFLU 17–0837**	***Ostrya carpinifolia***	**Italy**	**MH337873**	**MH337874**	**MH337875**
*Valsaria robiniae*	CBS 121890	VC	WU 33472	*Hippocrepis emerus*	Slovenia	KP687851	KP687851	KP687926
*Valsaria robiniae*	CBS 128015	VCA	WU 33474	*Caragana arborescens*	Austria	KP687853	KP687853	KP687928
*Valsaria robiniae*	CBS 125583	VCo1	WU 33475	*Colutea arborescens*	Austria	KP687855	KP687855	KP687930
*Valsaria robiniae*		V1R	WU 33477	*Robinia pseudacacia*	Italy	KP687869	KP687869	KP687944
*Valsaria robiniae*	CBS 139063	VIR1	WU 33478	*Robinia pseudacacia*	Italy	KP687870	KP687870	KP687945
*Valsaria rudis*		VQC	WU 33483	*Quercus cerris*	Croatia	KP687877	KP687877	KP687951
*Valsaria rudis*	CBS 139065	VQM	WU 33484	*Quercus macrolepis*	Greece	KP687878	KP687878	KP687952
*Valsaria rudis*	CBS 139066	VQP	WU 33485 (E)	*Quercus pubescens*	Austria	KP687879	KP687879	KP687953
*Valsaria rudis*		V3	WU 33486	*Quercus cerris*	Italy	KP687836	KP687836	KP687914
*Valsaria rudis*		V7	WU 33487	*Quercus petraea*	Austria	KP687844	KP687844	KP687920
*Valsaria rudis*		V31	WU 33488	*Quercus petraea*	Austria	KP687838	KP687838	KP687916
*Valsaria spartii*	CBS 139070	V6	WU 33505 (E)	*Spartium junceum*	Italy	KP687843	KP687843	KP687919
*Valsaria spartii*		V18	WU 33515	*Ulex parviflorus*	Spain	KP687823	KP687823	KP687901
*Valsaria spartii*		V19	WU 33516	*Teline linifolia*	Spain	KP687824	KP687824	KP687902
*Valsaria spartii*		V20	WU 33517	*Cytisus baeticus*	Spain	KP687826	KP687826	KP687904
*Valsaria spartii*		V21	WU 33518	*Retama monosperma*	Spain	KP687827	KP687827	KP687905

### Taxonomy

**Fig 3:**
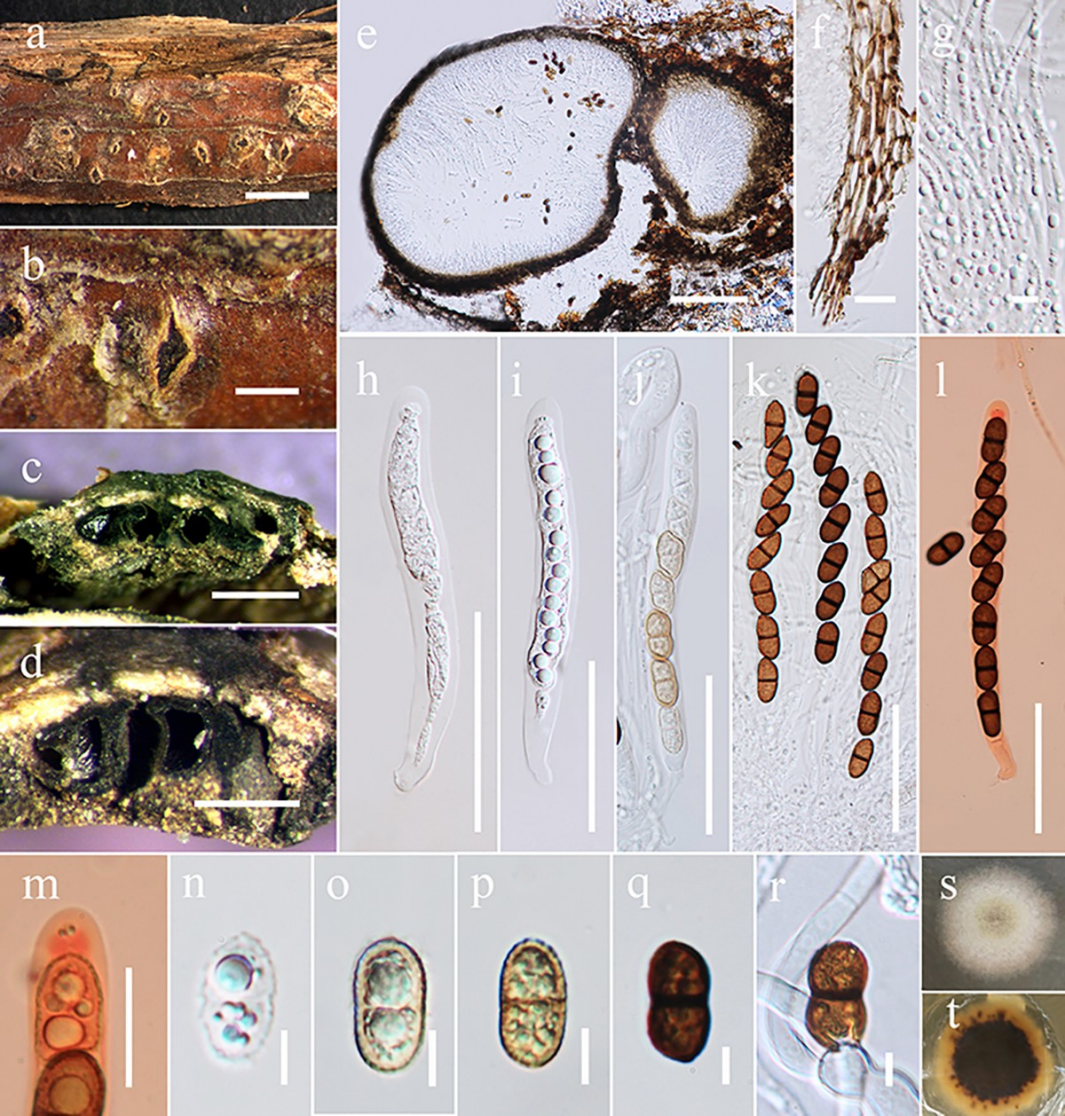
*Valsaria ostryae* (MFLU 17–0837). a-d Habit and appearance of ectostromata on host surface. e Vertical section of stroma. f Peridium wall. g Apically free pseudoparaphyses. h-j Immature asci k Mature asci. l, m Apical rings in Congo red. n-p Immature ascospores q Mature ascospore r Germinating ascospores s, t Culture characters on MEA (s above view, t back view). Scale bars: a = 500 μm, b = 200 μm, c,d = 500 μm, e = 200 μm, f = 10 μm, g = 5 μm, h-l = 50 μm, n-r = 5 μm.

***Valsaria ostryae*** D. Pem, R. Jeewon, Camporesi & K.D. Hyde, *sp*. *nov*. Fig 3

[urn:lsid:indexfungorum.org:names:554758]

Facesoffungi number: FoF 04614

Etymology–Name reflects the host from which the fungus is isolated.

Holotype–MFLU 17–0837

**Fig 4:**
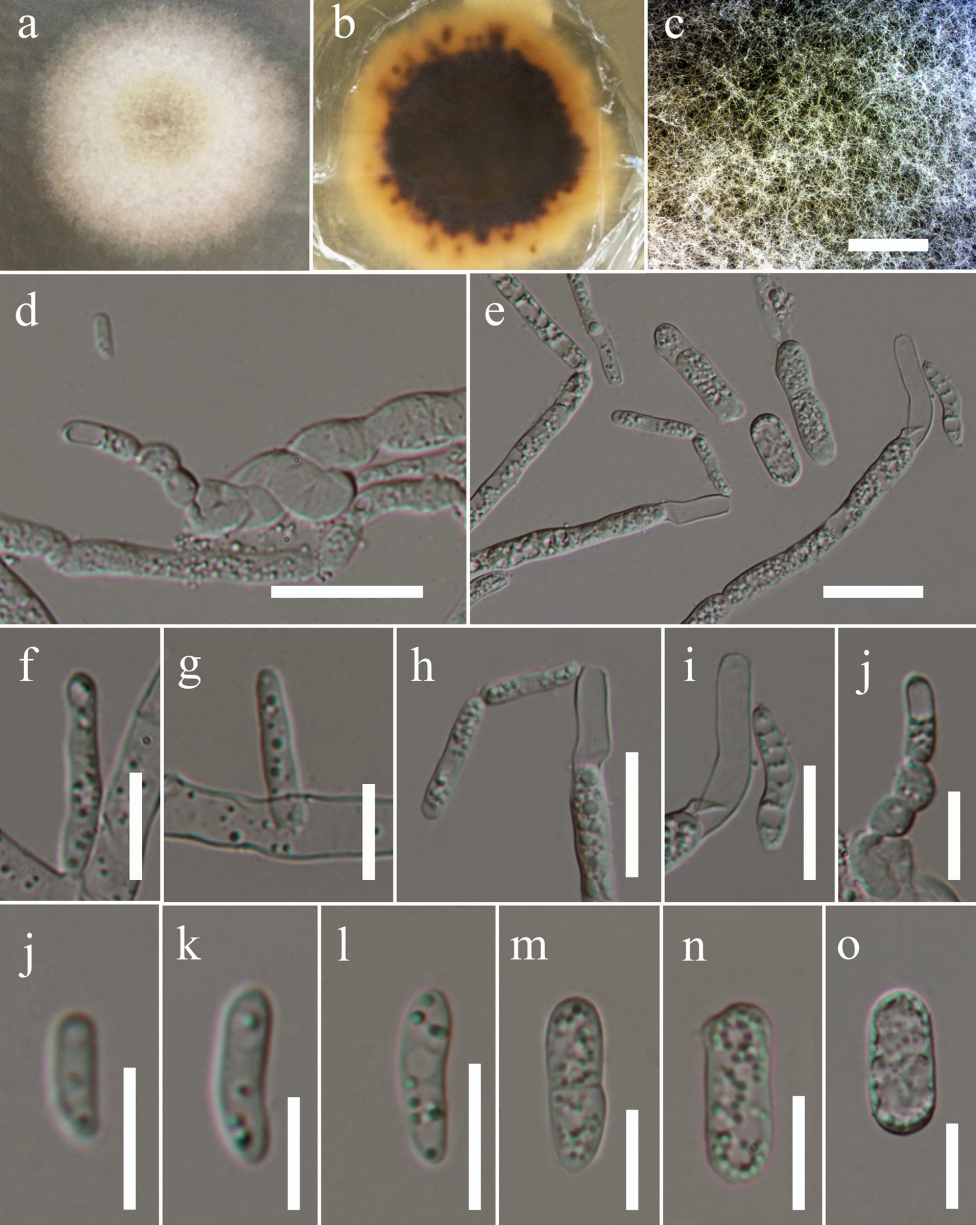
*Valsaria ostryae*: Asexual morph (MFLU 17–0837). **a-c Culture characteristics and mycelium. d,e hyphae with denticles and conidia (partly budding). f-j Phialides. j-o Conidia. Scale bars: c = 1000** μ**m, d = 20** μ**m, e = 10** μ**m, f-j = 10** μ**m, j-o = 10** μ**m.** Notes–*Valsaria ostryae* resembles *V*. *insitiva* (type species), in having pseudostromatic stromata, pseudoparenchymatous ectostroma, bitunicate asci containing short cylindrical ring and ellipsoid, dark brown ascospores. However, *V*. *ostryae* differs from *V*. *insitiva* in having narrower pseudoparaphyses (2–3 μm vs. 1.5–5 μm), guttule number in each ascospore cell (0 vs. 2–3) and in ornamentation and constriction of the ascospores. The ascospores are deeply constricted at septum and finely warted to reticulate in *V*. *ostryae* while those of *V*. *insitiva* are not or hardly constricted at the septum and finely tuberculate. Our multi-gene phylogeny suggests that *V*. *ostryae* is related to *V*. *lopadostomoides* and *V*. *rudis* but phylogenetically segregated from them with strong bootstrap support (100% ML, 100% MP, 1.00 PP). *Valsaria ostryae* has smaller stromata (500–800 μm high, 1000–1500 μm diam v.s 1200–1400 μm high, 1700–2500 μm diam), shorter asci (82–131 × 9–12 μm v.s (112–)115–128(–136)×(10.5–)11.0–14(–15.5) and have different ascospore ornamentation (finely warted to reticulate v.s distinctly warted, reticulate or spotted). Our new taxon is also morphologically different from *V*. *rudis* in having smaller stromata1000–1500 μm diam v.s 1200–1700 μm high, 1200–2500 μm diam) and smaller ascomata (277–479 μm high, 284–603 μm diam, v.s 3000–6000 μm high, 2000–5000 μm diam), Furthermore, a comparison of 600 nucleotides across the ITS (+5.8S) gene regions between *V*. *ostryae* and *V*. *rudis* reveals 5.31% base pair differences and that of *V*. *ostryae* and *V*. *lopadostomoides* reveals 6.60% base pair differences respectively. Hence, *V*. *ostryae* is introduced as a new species in the genus *Valsaria*.

Saprobic on dead branch of *Ostrya carpinifolia*. **Sexual morph**: *Stromata* pseudostromatic, erumpent from bark, scattered, sometimes aggregating in groups of 2–3; pustular, conical to subglobose with flattened base, 500–800 μm high, 1000–1500 μm diam., enclosed on top and/or at sides by a black, 20–40 μm thick pseudoparenchymatous crust. *Ectostroma* often forming sub- or inversely stellate structures of 3–5 greyish, brown to black, tubercular segments; tissue beneath crusty pseudoparenchymatous; tissue between necks and at stromatal base prosenchymatous, grey, soft, mixed with bark cells. Ostiolar openings inconspicuous at the surface or indistinctly papillate. Ascomata arranged in valsoid configuration, monostichous, subglobose to flask-shaped, 277–479 μm high, 284–603 μm diam., peridium 25–40 μm thick, of pale brown compressed cells of *textura angularis*. *Paraphyses* numerous, unbranched, tapering upwards, apically free, 2–3 μm wide. *Asci* 82–131 × 9–12 μm ($\bar{x}$ = 110 × 11 μm, n = 15), 8-spored, bitunicate, fissitunicate, cylindrical, apex containing an ocular chamber and a short cylindrical to barrel-shaped ring, 2–3 μm wide, 3–4 μm high ($\bar{x}$ = 2 × 3 μm, n = 10), staining in Congo Red. *Ascospores* 16–17 × 7–9 μm, ($\bar{x}$ = 16 × 7 μm, n = 20), ellipsoid, 2-celled, dark brown, with a dark, thick, central, slightly constricted septum; surface finely warted to reticulate. **Asexual morph**: *Pycnidia* 0.1–0.4 mm diam, globose, multiloculate, black, irregularly surrounded by white mycelium, wall pseudoparenchymatous, thick-walled, globose to clavate cells, giving rise to short, cylindrical to ellipsoid hyaline cells. *Phialides* 7–18 × 2–5 μm ($\bar{x}$ = 13 × 3 μm, n = 20) produced in more or less parallel, densely packed in palisades, narrow, lageniform, cylindrical or conical. *Pycnoconidia* 14–18 × 2–9 μm ($\bar{x}$ = 16 × 5 μm, n = 20) oblong, cylindrical to ellipsoid, sometimes subglobose, 1-celled, hyaline, smooth, lower end sometimes truncate (Fig 4).

Culture characteristics: Ascospores germinating on MEA within 24 h. Colonies growing on MEA, reaching 2 cm diam. in 1 week at 16°C. Mycelium medium dense, circular, slightly raised, surface smooth, edge slightly crenate, thinly hairy, slightly irregular margin, above whitish to pale yellow, reverse dark brown in middle and yellowish at the edge.

Material examined: ITALY, Province of Forlì-Cesena [FC], Santa Sofia, Camposonaldo, on dead branches of *Ostrya carpinifolia* (*Betulaceae*), 18 March 2017, E. Camporesi, IT 3290 (MFLU 17–0837, **holotype**; HKAS 97468, isotype) ex-type living culture MFLUCC 18–1123, ICMP 22558.

## Discussion

Species of *Valsaria* occur primarily on woody parts of ornamental plants but is also distributed on a wide range of hosts. Our new taxon was isolated from *Ostrya carpinifolia* which belongs to Eudicots and Betulaceae family [20]. Substrates colonised by members of *Valsaria* recorded until now are all dicotyledons of the Fabaceae, Fagaceae and Vitaceae. *Valsaria lopadostomoides* can be found on evergreen oaks, such as *Quercus ilex* (Fagaceae) while *V*. *rudis* occur on deciduous oaks, such as *Quercus cerris* and *Q*. *petrae* (Fagaceae). Woody plant hosts of *V*. *lopadostomoides* and *V*. *rudis* are closely related to each other as both are accommodated in the Fagaceae which is a monophyletic family close to Quercoideae [21]. *Valsaria neotropica* has been isolated from unidentified hosts in the Neotropics and seems to be distributed in the tropical terrestrial ecoregions of the Americas and the South American temperate zone. *Valsaria insitiva* was described mainly from non-fabaceous hosts and *Vitis* (Vitaceae) which is accommodated in its own order Vitales and closely related to Rhamnaceae in the order Rhamnales [22]. *Valsaria robiniae* have been reported from *Amorpha*, *Caragana*, *Colutea*, *Hippocrepis* and *Robinia* species in Central Europe, submediterranean regions and Eastern USA while *V*. *spartii* occur on various species of woody Fabaceae in the Sub-Mediterranean. Host distribution of *V*. *robiniae* and *V*. *spartii* seems to be related as most of the species are from Fabaceae which is the third largest family of angiosperms after Orchidaceae and Asteraceae [23]. Based on available literature, it is apparent that *Valsaria* has a wide host range and species are less likely to be host specific. As far as we are aware, no *Valsaria* species are known from *Ostrya carpinifolia*. We compare the new species with *V*. *insitiva*, the type species of *Valsaria* and they share similar characters. However, they differ in the number of guttules in each ascospore cell, as well as in the surface ornamentation of the ascospores (Fig 3). Our new taxon can also be compared to *V*. *sparti* and *V*. *robiniae* and differs in ascospores that are not constricted. *Valsaria ostryae* also shares similar morphological characters to *V*. *rudis* and has 2–3 guttules in each ascospore cell, while *V*. *rudis* has no guttule in its ascospore cells. Previous morphological studies and phylogenetic investigations based on SSU, ITS-LSU RPB2 and TEF genes data have provided insights into the taxonomy and classification of *Valsaria* and its allies [1]. The most recent taxonomic work dealt with more than 100 collections of *Valsaria sensu lato*, including two new species, which has helped to resolve generic delimitation, circumscription and classification of *Valsaria* [1]. In this study, a new species with a unique morphological character is illustrated and its relationships with other species are investigated based on multi-gene phylogenetic analyses. Our molecular data analyzed herein generated phylogenies that are congruent with those of Jaklitsch et al. [1] and helps to resolve intergeneric relationships between *Bambusaria*, *Myrmaecium* and *Valsaria*. Three well supported monophyletic groups are recovered which support *Bambusaria*, *Myrmaecium* and *Valsaria* as phylogenetically distinct genera (Fig 2). Nevertheless, we observe that several morphological characters such as ostiole, apical ring, and pseudoparaphyses are interspersed in several clades which could be examples of homoplasy in morphological characters within the *Valsariaceae*. Our molecular phylogeny provides an overview of the usefulness of specific morphs for species and generic delineation.

One interesting finding herein is the significance of spore surface ornamentation in taxonomy. Ornamentation is a character commonly used to delineate species [24–28]. The taxonomy of fungi is based to a large extent on spore characteristics including spore size, shape, color, surface ornamentation and ontogeny [29–34]. Among asexual fungi, spore pigmentation and ornamentation have been crucial in the taxonomy of appendaged coelomycetes. Jeewon et al. [35, 36], Maharachchikumbura et al. [37] and Liu et al. [38] have demonstrated that versicolourous and nonversicolourous spore can be used to segregate species. Among Pleosporalean fungi, Punithalingam [39] used spore surface ornamentation as a diagnostic feature to differentiate *Coniothyrium* species. Among other sexual fungi, twenty-one species of *Eupenicillium* were successfully segregated into two groups (with v/s without distinct equatorial ridges) based on ornamentation of ascospores [40]. Spore septation and colour have also been used as characters for distinguishing genera and species of the family *Lophiostomataceae* by many mycologists such as Saccardo, who established 9 separate genera using these criteria [41]. Species of the genus *Aspergillus* Section *Nigri*, which are taxonomically confused and complex have also been identified and delineated based on spore ornamentation [42]. The latter has also been reported to be useful to demarcate sexual and asexual species at the familial and generic level [43–47]. Surface ornamentation and wall development have also been examined to segregate species of the Pachyphlodes (Pezizaceae, Pezizales) as well as to differentiate between species, genera and families of the Basidiomycetes [48].Villegas et al. [49] reported spore ornamentation as a useful character in differentiating various genera within Gomphales. *Clitopilus reticulosporus* was described as a new species (Entolomataceae, Agaricales) based on its unique spore ornamentation and the spore evolution theory has been well discussed by Morgado et al. [50]. Interestingly, ornamentation of ascospores as well as guttule number have also been significant to establish new genera and demarcate taxa within Helvellaceae (Pezizales, Ascomycota) such as *Gyromitra* subgenus *Melaleucoides* (warted, non apiculate, biguttulate ascospores), *Gyromitra* subgenus *Caroliniana* (coarsely reticulate ascospores with multiple spicules at the poles) and *Helvella* subgenus *Cerebriformae* (globose, echinate ascospores) [51].

A similar phylogenetic scenario is reported herein. The three clades corresponding to different genera can be easily distinguished based on spore ornamentation. *Valsaria* is characterized by fine warted to reticulate or finely tuberculate ascospores surface, whereas *Bambusaria* and *Myrmaecium* species possess densely reticulate and longitudinally ribbed ornamentation respectively. Even at the species level, ornamentation can be used to a certain extent to segregate species. For example, *V*. *insitiva* the type species, can easily be delineated from other *Valsaria* species herein, except *V*.*neotropica*, based on finely tuberculate spore ornamentation. *Valsaria rudis* and *Valsaria lopadostomoides* are similar in having ascospores surface ‘warted to reticulate’ but additionally the ascospores of *V*. *lopadostomoides* is described as ‘spotted’ [1]. In addition, *M*. *fulvopruinatum*, characterized by densely reticulate spores can be distinguished from *M*. *rubrum* and *M*. *rubricosum*, whose spore surface has labyrinth ornamentation. We can therefore rely mostly on such morphology to circumscribe species at this taxonomic level. However, relationships based on other morphs, such as stromatal characteristics, presence or absence of ostioles/ pseudoparaphyses/apical ring, septum constriction and number of guttules show little congruence with findings from molecular data and are not phylogenetically significant for intergeneric segregation. Nevertheless some of these characters, especially the latter two, if used judiciously can be used to circumscribe species. For instance, *V*. *lopadostomoides* and *M*. *fulvopruinatum* can be distinguished from other *Valsaria* and *Myrmaecium* species on the basis of number of guttules. The number of guttules per spore has also been considered as a diagnostic feature to delineate taxa at the intraspecific level. For example, guttule character was found as important to separate species and varieties recognized in the genus *Phoma* [52] and helped to provide a key to differentiate among thirty taxa. Recently, Southworth et al. [53] demonstrated that number of guttules in each spore has been useful to delineate species of *Balsamia* when other characters overlap. Vander [54] reported that the occurrence of large guttules was useful to distinguish species of *Phyllosticta*, especially when used in combination with other characters. Presence of guttule is especially useful when confirming diagnosis of herbarium material from fresh specimen and this has helped to validate *Lophiostoma diminuens* (Pers.) Fuckel [55]. The diagnostic microscopical characters supporting ranks of different taxa of the family Sarcoscyphaceae were mainly based upon differences in the guttule (lipid) number and pattern of living ascospores [41]. To differentiate genera within *Gnomoniaceae*, Monod [56] used characters such as presence or absence of small guttules. The latter has also been considered as a diagnostic feature to separate *Diaporthe* species from grapevine [57] as well as freshwater fungal species [58, 59]. Guttule characters can also be used to distinguish our new taxon, *V*. *ostryae* from others. Phylogeny herein indicates *V*. *ostryae* to be more closely related to *V*. *lopadostomoides* and *V*. *rudis* than any other species. Although these three species share several common morphological features (pseudostromatic stromata, apically free pseudoparaphyses, apical ring and dark brown ascospores), our new taxa is clearly distinct in number of guttules and surface ornamentation.

To further substantiate establishment of *Valsaria ostryae* as a new species, the complete ITS and RPB2 gene regions were compared. Nucleotides differences reveal clear cut differences that warrant new species status. For example, *V*. *ostryae* differs from *V*. *lopadostomoides* by 5.31% bp difference in the ITS gene regions. Considering both molecular and morphological data we regard *V*. *ostryae* as a new taxon that warrants species rank. The genus *Valsaria* is known to comprise taxa with minor morphological variations that result in taxonomic confusion. For example, the ascomata of *Valsaria insitiva*, *V*. *lopadostomoides* and *V*.*neotropica* are monostichously arranged in valsoid configuration measuring 250–450 μm high, 180–400 μm diam, peridium 14–25 μm thick, cylindrical asci with a short cylindrical to barrel-shaped ring measuring in between 96–112 μm × 11–18.5 μm and 2-celled, ellipsoid dark brown ascospores with the size ranging from 12–19 μm × 6.5–11.7 μm. However, when analyzing the gene regions, they are all genetically different. ITS base pair difference between *Valsaria insitiva* to *V*. *lopadostomoides* is 2.4%, *V insitiva* to *V*. *neotropica* is 11.8%, and *V*. *lopadostomoides* to *V*. *neotropica* is 14.3%. Likewise, other *Valsaria* species such as *V*. *robiniae*, *V*. *rudis*, *V*. *spartii* and our new taxon share similar morphological characters which are not really meaningful in species delineation.

This study provides delightful taxonomic insights across *Valsaria* species. We anticipate that taxonomists will pay particular attention to these characters that can potentially aid in species identification of other genera. Our study also reveals that *Valsaria* could be a speciose genus and there is a need for more collections targeting a wide variety of hosts which can help to answer pertinent questions with regards to fungal diversity issues.
